# Studying the microbiome and its complexities: an interview with Alan Walker

**DOI:** 10.1186/s12915-018-0599-z

**Published:** 2018-11-01

**Authors:** Alan W. Walker

**Affiliations:** The Rowett Institute, University of Aberdeen, Foresterhill, Aberdeen, AB25 2ZD UK

**Keywords:** microbiome, personalised nutrition, diet, contamination, open access publishing

## Abstract

Alan Walker is a Senior Lecturer at the University of Aberdeen, UK, studying the intestinal microbiota and its interactions with the host’s diet. In this interview, Alan discusses his research interests, earlier studies of the ways contaminants can affect microbiome analyses, the excitement of experiments going well, and why science doesn’t need to be combative.

## What are the questions driving your research?

I am based at The Rowett Institute, which is a centre for inter-disciplinary nutrition research. Our work is therefore focussed on interconnections between what we eat, the gut microbiota, and subsequent impacts on host health. In order to develop more effective microbiota-targeted dietary interventions we need a much better understanding of which gut microbiota species might be beneficial for health, and in which ways. We also need to better understand how a person’s gut microbiota will respond to specific dietary interventions, and how this response can vary between different individuals.

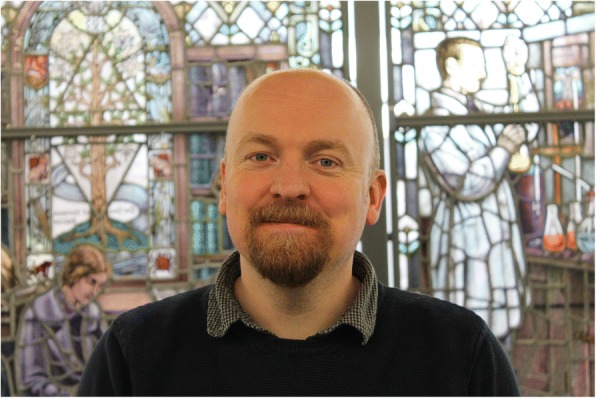



**Fig. 1** Alan Walker

## In your 2014 paper in *BMC Biology*, you were able to show that DNA contamination in lab reagents can lead to flawed microbiome analyses. Can you tell us a little bit about the impact of that study on microbiome research?

The problem of lab reagent contamination had been described many years previously [1]. However, the use of DNA-based approaches to profile microbial communities has increased hugely over the past decade, and it seemed that many groups were not fully aware of this problem. There were a number of published studies describing unexpected microbial communities in various environments, and many of the bacteria appearing in these samples overlapped with those we routinely found in our negative controls. Our study was therefore an attempt to properly demonstrate the scale of the contamination problem, to publicise it more widely to a new audience, and to describe measures that researchers could take to try and limit the impact.

Overall, the response to the paper in *BMC Biology* has been very positive. It was publicised widely, has been very well cited, and followed up by other studies that echo the concerns raised (e.g. [2]). Although some low biomass microbiome studies are still coming out that do not include appropriate negative controls, I would hope that greater awareness of the problem has prevented the publication of misleading results. At the very least, it has helped to generate more discussion about the limitations of DNA sequence-based methods, which can only be a good thing for the field of microbiome research.

## Looking back, is there a project that your lab pursued that stands out for you as particularly inspiring, tough, or simply memorable?

There have been perhaps a handful of occasions in my life as a scientist when a set of experimental results came back and they were so compelling that I wanted to jump for joy. A personal favourite is perhaps the first study I ever did during my PhD, which established that pH can have major impacts on the metabolic outputs from colonic microbial communities [3]. I stood and waited by the old gas chromatography machine as it gradually fed out results, which showed that the observations from the initial pilot experiment had been replicated perfectly in the second. Lovely.

## Is there a paper or a scientist that inspired you, or was seminal for your research?

I have been very lucky in my career to have worked with lots of brilliant people, including supervisors, co-workers, students and collaborators. To a greater or lesser degree, all of them have inspired my research. If I had to pick out one paper that I often go back to, I thoroughly recommend a review by John Cummings and George Macfarlane from 1991 [4]—it serves as an excellent reminder that gut microbiome research has a rich history that extends far beyond the advent of next generation sequencing!

## What are your guiding principles for running a lab? Do you have any advice to share with our readers?

I am still relatively new to running a lab (the *BMC Biology* paper on contamination was my first as senior author), but my main guiding principle in science has always been to work with interesting and inspiring people. Science does not have to be combative. I’m always staggered to meet scientists who want to keep everything in house, as interdisciplinary collaboration is one of my favourite things about the job. So, I guess my main piece of advice is to be open to collaborative work, and then to be nice to each other! It’s critically important to ensure that there is mutual respect and due credit given between all contributing partners.

## If you could, what would you tell your younger self?

It’s important to acknowledge the role that luck plays in a successful scientific career, so do your best to make the most of opportunities that arise. Oh, and learn R. You won’t have proper time for this when you are running a group, and will bitterly regret your over-reliance on Excel…

## What are the open questions you’d like to see addressed in your field?

See the answer to “What are the questions driving your research?”! I believe that, moving forward, the gut microbiome will need to be integrated more extensively (and effectively) into the emerging field of personalised nutrition.

## What kind of innovations in publishing would you like to see happen?

Given the amount of misinformation about the microbiome that is currently available in the popular press, and on the internet, I believe that it is important that the general public be given more opportunity to see and judge the underlying science, should they so wish.

As such, I would like to see the final, copy edited versions of all peer reviewed manuscripts being fully open access to the public. For those of us without a budget to cover open access charges, this is not always possible, but it is certainly something to aspire to. I would encourage any efforts to facilitate this process.

I would also be in favour of mandatory open access lay summaries, scrutinised by peer review at the same time as the scientific manuscript, appearing online alongside research articles. The potential benefits have been well covered by others [5].

**Website:** https://www.abdn.ac.uk/rowett/research/dr-alan-walker-643.php

**Twitter:** @_Faecal_Matters
